# Devil’s claw (*Harpagophytum procumbens*): is the buzz in Google justified?

**DOI:** 10.1007/s00210-025-03974-7

**Published:** 2025-03-05

**Authors:** Finn Erik Bargsten, Roland Seifert

**Affiliations:** Institute of Pharmacology, Hannover Medical School, Carl-Neuberg-Str. 1, 30625 Hanover, Germany

**Keywords:** *Harpagophytum procumbens*, Devil’s claw, Product analysis, Study analysis

## Abstract

Chronic muscle and joint pain affect many people. However, current treatment options harbor a considerable risk of adverse drug reactions, which is why the search for other treatment alternatives is constantly expanding. As a result, research is also focusing on plant-based products. These include devil’s claw, a medicinal plant from the sesame family, which is native to the African continent and is used there as a traditional remedy. In this study, 16 different criteria were selected for a critical analysis of devil’s claw products, which included aspects such as drug-drug interactions, adverse drug reactions, and recommended daily dose, and 88 products were analyzed with the help of these criteria. Furthermore, products classified as food supplements, dietary supplements, and herbal medicinal products were compared with each other. Our goal was to analyze how consumers are informed, with a focus on consumer safety. The primary objective was not to analyze the actual ingredients of the products or to compare them based on their composition. The results almost invariably show the pharmaceutical reliability of herbal medicinal products. In contrast, products marketed as food supplements and dietary supplements have questionable reliability, as most products in these categories allow manufacturers more freedom and impose fewer controls and requirements. Devil’s claw products labeled as food or dietary supplement show significant gaps in consumer information compared to herbal medicinal products. Product naming and packaging are often suggestive, potentially misleading consumers. Our study shows that food supplements and dietary supplements are clearly pursuing financial interests, and that consumer protection is often neglected in the process. The study analysis also revealed a limited number of clinical studies, no long-term studies, and poor methodological quality, undermining their reliability.

**Supplementary Information:**

The online version contains supplementary material available at 10.1007/s00210-025-03974-7.

## Introduction

Devil’s claw refers to a group of plants belonging to the genus *Harpagophytum*, which includes the two species *Harpagophytum procumbens* (Burch.) DC. ex Meisn. and *Harpagophytum zeyheri* Decne. Both species are used interchangeably, as no superiority of one over the other has been established (Brendler 2021). In vivo studies have shown that both exhibit similar analgesic and anti-inflammatory properties (Baghdikian et al. 1997). For clarity, we will refer to HP in the following text.

HP has been known to people living in South Africa for centuries and has been used to treat various ailments. As a member of the sesame family (Pedaliaceae), this plant is primarily found in Namibia, Botswana, Zimbabwe, and South Africa. It owes its name to the hook-like outgrowths of its fruit. The secondary roots are use medicinally by drying and crushing them (EMA 2021). In traditional medicine in South Africa, it is mainly used for digestive problems, loss of appetite, and to relieve labor pains (EMA 2021). In Europe, the focus is more on painful arthritis, tendinitis, loss of appetite, and dyspeptic complaints (EMA 2021). Its ingredients include iridoid glycosides, flavonoids, and large quantities of water-soluble carbohydrates. The iridoid glycosides also include harpagoside (Fig. S10) and harpagide (Fig. S11). In vitro experiments indicate that harpagosides and harpagide inhibit COX-2. Harpagoside was found to be a moderately potent ligand at a G protein-coupled receptor, a nuclear receptor ligand, protease inhibitor, and enzyme inhibitor, whereas harpagide turned out to be an enzyme inhibitor, G protein-coupled receptor ligand, ion channel modulator, nuclear receptor ligand, and protease inhibitor (Rahimi et al. 2016) but are also able to reduce concentrations of TNF-α, IL-6, and IL-8 in a concentration-dependent manner (Hostanska et al. 2014).

However, in vitro and in vivo animal models in which isolated harpagoside was used showed that this substance had little or no anti-inflammatory effect (EMA 2021). In contrast, devil’s claw extracts showed a significant anti-inflammatory effect. This suggests that harpagoside is not the only active or main active substance contributing to the claimed anti-inflammatory effect of HP (EMA 2021).

**Fig. 1:**
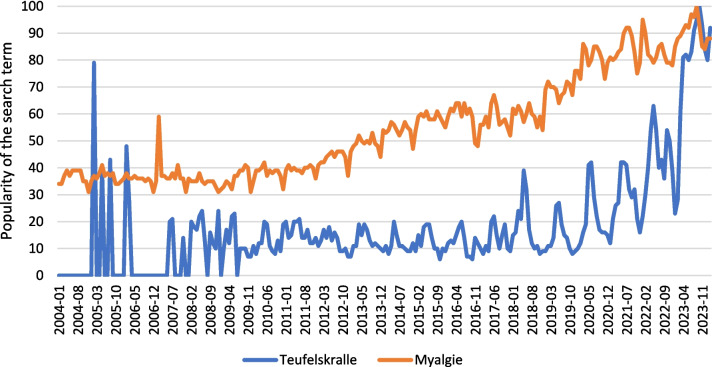
Development over time of Google searches for “Teufelskralle” (devil’s claw) and “Myalgie” (myalgia) (Teufelskralle: https://trends.google.de/trends/explore?date=all&q=%2Fm%2F08s3vx&hl=de, accessed 02 March 2024; Myalgie: https://trends.google.de/trends/explore?date=all&q=%2Fm%2F013677&hl=de, accessed 02 March 2024)

Osteoarthritis is the most widespread joint disease not only in Germany but also worldwide (Woolf and Pfleger 2003). As a result, herbal alternatives, such as DS, FS, and HMP (Table 1) made from devil’s claw, are increasingly becoming the focus of general interest. Figure 1 shows the increasing public interest in devil’s claw and its possible effects and myalgias, as the Google search shows. Since conventional anti-inflammatory drugs, such as COX inhibitors, are associated with possible adverse drug reactions that cannot be ignored when taken over a longer period of time (Bindu et al. 2020), there is a demand for alternatives.

**Table 1:** Showing a summarized explanation of HMP, FS, and DS

Category	Characteristics
HMP (https://www.ema.europa.eu/en/medicines/herbal/harpagophyti-radix. accessed 08.08.2024)	• Regulated by the EMA in the EU • In the EU, HMP are authorized as traditional herbal medicinal products if there is insufficient evidence of their clinical efficacy • Prerequisite: Use for at least 30 years, including at least 15 years in the EU, and must be intended for oral use without medical supervision • In this case, the long-standing medicinal use can demonstrate plausibility of efficacy in accordance with the Traditional Herbal Medicinal Products Directive (2004/24/EC) • Quality requirements, e.g., “good manufacturing practice” • For authorization, the manufacturer must submit an application to the competent national authority
FS (https://www.efsa.europa.eu/de/topics/topic/food-supplements, accessed 08.08.2024)	• Regulated by EFSA in the EU in accordance with the EU General Food Law • Are classified as foods but are intended to supplement and not replace the normal diet • May not advertise to treat or cure certain diseases • However, the manufacturer is responsible for the quality and safety of their product • Only the addition of minerals and vitamins, the forms in which they may be used and the substances which may not be contained are explicitly regulated • No legally defined maximum quantities for ingredients • Label must contain the following: A recommended daily intake in portions, information that the recommended daily dose must not be exceeded, that the products must be kept out of the reach of small children, and that FS should not be used as a substitute for a varied diet
DS (https://www.fda.gov/food/information-consumers-using-dietary-supplements/questions-and-answers-dietary-supplements, accessed 08.08.2024)	• Regulated by FDA in the USA in accordance with “The Dietary Supplement Health and Education Act of 1994” (DSHEA) • DS are considered a subgroup of foods • Ingredients are divided into two categories, dietary ingredients and new dietary ingredients • Claims regarding structure/function, a nutrient deficiency disease benefit, or a general wellness claim on the label, the manufacturer must demonstrate that the claim is truthful or not misleading • Label must also include a disclaimer stating that the FDA has not evaluated the claim as well as that the product is not intended to diagnose, treat, cure, or prevent any disease, the term DS or the word "supplement" in connection with the product name and all ingredients • All DS must be swallowable

**Fig. 2:**
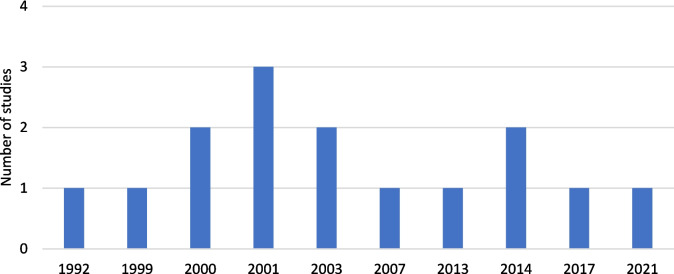
Time trend of clinical trials on devil’s claw on PubMed (https://pubmed.ncbi.nlm.nih.gov/?term=devil%27s+claw&filter=pubt.clinicaltrial, accessed 02 March 2024)

In contrast to the increasing public interest, only a few clinical studies can be found on PubMed (Fig. 2). Although a pain-relieving effect has been observed in clinical studies investigating the effects of devil’s claw on joint pain, the EMA assessed the clinical trials as being of insufficient quality as many studies have different study designs and the majority are not randomized and placebo controlled (EMA 2021). Furthermore, no meta-studies have yet been conducted on this topic. Nevertheless, the traditional use of the plant and that it has been used safely provide evidence of a plausibility of efficacy (EMA 2021).

The research gaps, including limitations in the design of existing studies and the scarcity of clinical trials (Fig. 2), combined with the growing public interest in devil’s claw as a phytotherapeutic agent (Fig. 1), reveal a notable discrepancy. This has motivated us to investigate the market for devil’s claw products and their properties in greater detail. Our analysis focused on determining the type and extent of information provided by manufacturers to consumers and patients in relation to the HMP. We wanted to assess how comprehensively and transparently relevant details were communicated. However, the products included in this analysis were not analyzed for their individual ingredients or components, as the focus was solely on the availability and quality of consumer information rather than a detailed compositional analysis.

In this study, we analyzed 88 devil’s claw products, consisting of FS, DS, and HMP, according to 16 different criteria as well as 7 clinical studies to gain an overview of the current market and study situation.

## Material and methods

In order to gain an overview of the current market situation of devil’s claw products, this theoretical study analyzed products according to various criteria and conducted research on the current study situation. The data were collected between 06.2022 and 05.2023. The websites Shop-Apotheke, DocMorris, iHerb, Carethy, Piping Rock, and Walmart were used to search for the products. The keywords “Teufelskralle,” “Devil's claw,” and “*Harpagophytum procumbens*” were used in the search. The selection of websites included products that are available on the European and US markets. The analysis included products whose ingredients included devil’s claw or its extracts. To obtain information, the package inserts were used for HMP, and the information was provided by the manufacturer for DS, FS, and cosmetic products. To analyze the devil’s claw products, 16 different criteria were selected to obtain as differentiated a picture as possible. In addition, the meta-database PubMed was searched using the keywords “Teufelskralle,” “Devil’s claw,” and “*Harpagophytum procumbens*” for current, clinical full-text studies. The analyzed data were summarized in tabular form, and the selected studies were also analyzed according to evidence level. The chemical structures of harpagosides and harpagides were generated using MolView (https://molview.org, accessed January 21, 2025).

The most important data obtained in this study are shown within the main text; supplemental information is presented in Figs. S1, S2, S3, S4, S5, S6, S7, S8, S9, S10, and S11.

### Analysis of devil’s claw products

A total of 88 HMP, FS, and DS were analyzed according to 16 different criteria, and the results were recorded in an Excel table (Table 2). The criteria used consisted of classification, prescription type, dosage form, indications/scopes of application and contraindications for use, adverse drug reactions, possible drug-drug interactions, recommended daily dose, recommended maximum duration of use, age limit, pictograms on the packaging of the products as well as their size, daily price, active and additional ingredients, and the alleged effects. Finally, the data from the Excel table was converted into diagrams.

**Table 2:** Criteria with which the products were analyzed

Analysis criteria	Explanation
Classification	On which legal basis the product was marketed
Alleged effects	Effects that the product is supposed to have, according to the manufacturer
Indications/scopes of application	Indications or scopes of application for which, according to the manufacturer, the product should be taken
Contraindications	Contraindications for which, according to the manufacturer, the product should not be taken
Adverse drug reactions	Adverse drug reactions which may occur when taking the product, according to the manufacturer’s instructions
Drug-drug interactions	Interactions which, according to the manufacturer, may occur between other medicines or substances and the product
Dosage form	The way in which the product should be taken according to the manufacturer’s instructions
Recommended daily dose	Quantity in which the product should be taken according to the manufacturer’s instructions
Recommended maximum duration of use	Recommended maximum period of time during which the product may be taken according to the manufacturer’s instructions
Package size	Quantity in which the product is sold
Daily price	Costs that arise if the daily intake complies with the manufacturer’s recommendations or specifications
Number of active ingredients	Number of active ingredients to be taken from the manufacturer’s instructions
Number of additional ingredients	Number of additional ingredients to be taken from the manufacturer’s instructions
Prescription type	Whether the product is available over the counter or only with a prescription
Age limit	The age at which the product can be taken, according to the manufacturer’s instructions
Pictograms	Illustrations shown on the packaging of the product

#### Classification

Under the aspect of classification, it was analyzed how the products were offered for sale or to which legal basis they were assigned, e.g., FS or medicinal products.

#### Prescription type

This criterion includes whether the products were freely available for sale or could only be purchased on presentation of a prescription issued by a doctor.

#### Dosage form

The form in which a product should be taken is analyzed using the criterion of dosage form.

#### Indication/scopes of application

The criterion includes the indications for the respective HMP and the scopes of application for the products that are not subject to the pharmaceutical law. In cases where no clear indications or scopes of application were found for the respective products, this was summarized as “care.”

#### Contraindication

The contraindications specified by the respective manufacturer were analyzed under this criterion.

#### ADR

It was checked whether a product provided information on possible adverse drug reactions.

#### Drug-drug interactions

This criterion was used to check whether possible drug-drug interactions with other medicinal products, foodstuffs, or similar were stated.

#### RDD

Under recommended daily dose, the daily dose recommended by the manufacturer was analyzed. If the manufacturer gave a margin of discretion regarding the frequency of intake, a mean value was calculated from the maximum and minimum values. If the calculated mean value was between two figures, it was rounded up. The recommended frequency of intake was then multiplied by the single dose.

#### Daily price

How much money the products cost in daily use is analyzed using the daily price criterion. If no RDD was given by the manufacturer, the lowest possible application was used to calculate the daily price, e.g., one tablet or one stroke of the liquid product. In order to be able to calculate the daily prices equivalently, a stroke was standardized to 2 ml for liquid products. A drop was standardized to 0.05 ml.

#### Recommended maximum duration of use

This criterion analyzes the period of use specified by the respective manufacturer for the corresponding product.

#### Pictograms

The illustrations on the packaging were analyzed using the pictograms criterion. In addition, the images were analyzed for a possible suggestive effect.

#### Package size

The size of the package was also analyzed. A distinction was made between products in tablet form, in liquid form, or in solid form, which were not offered in tablet form. Furthermore, for the sake of clarity, intervals were selected in the associated diagrams into which the package sizes were sorted.

#### Active ingredients and additional ingredients

The criterion of active ingredients analyzed how many of these were in the corresponding product. For the additional ingredients, those substances were analyzed that were found in the products in addition to the active ingredients. Intervals were selected in the diagrams for both criteria. As soon as no clear distinction was made by the manufacturer between active ingredients and additional ingredients, it was assumed that devil’s claw extract was the only active ingredient in the respective product.

#### Age limit

The age limit criterion was used to analyze for which age groups or from which age the manufacturers recommended the use.

#### Alleged effects

The effects claimed by the manufacturers for the products were analyzed in the criterion of alleged effects.

### Analysis of clinical studies

#### Search strategy

We used the PubMed database to search for clinical studies that have been published before May 2023. Therefore, we used the following keywords: “Teufelskralle,” “*Harpagophytum procumbens*,” and “Devil’s claw.”

#### Selection criteria

**Fig. 3:**
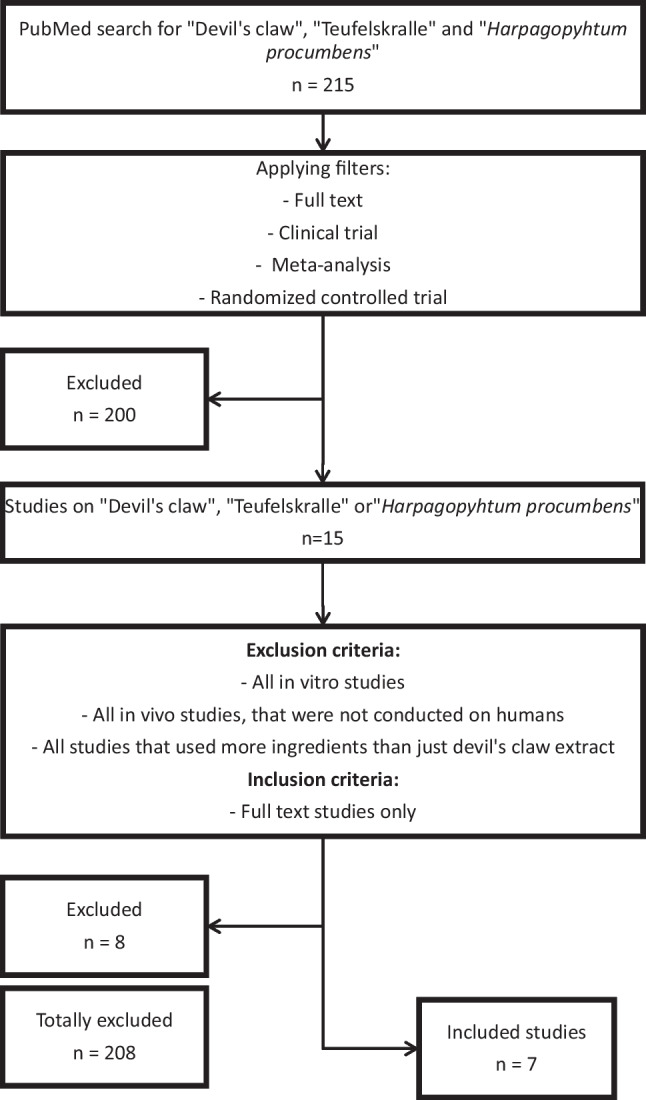
Procedure of the analysis of clinical studies

We found 215 results under the search terms mentioned. The filtering process is shown in Fig. 3. In the first step, all studies that could not be classified in any of the following filter categories were excluded: “full text,” “clinical trial,” “meta-analysis,” and “randomized controlled trial” (*N* = 200). In the second step, all in vitro studies and in vivo studies that were not conducted on humans were removed from the remaining studies (*N* = 15). Furthermore, all studies with preparations with more ingredients than just devil’s claw were excluded (*N* = 8). The clinical full-text studies still in question (*N* = 7) were analyzed (Table 3).

**Table 3:**
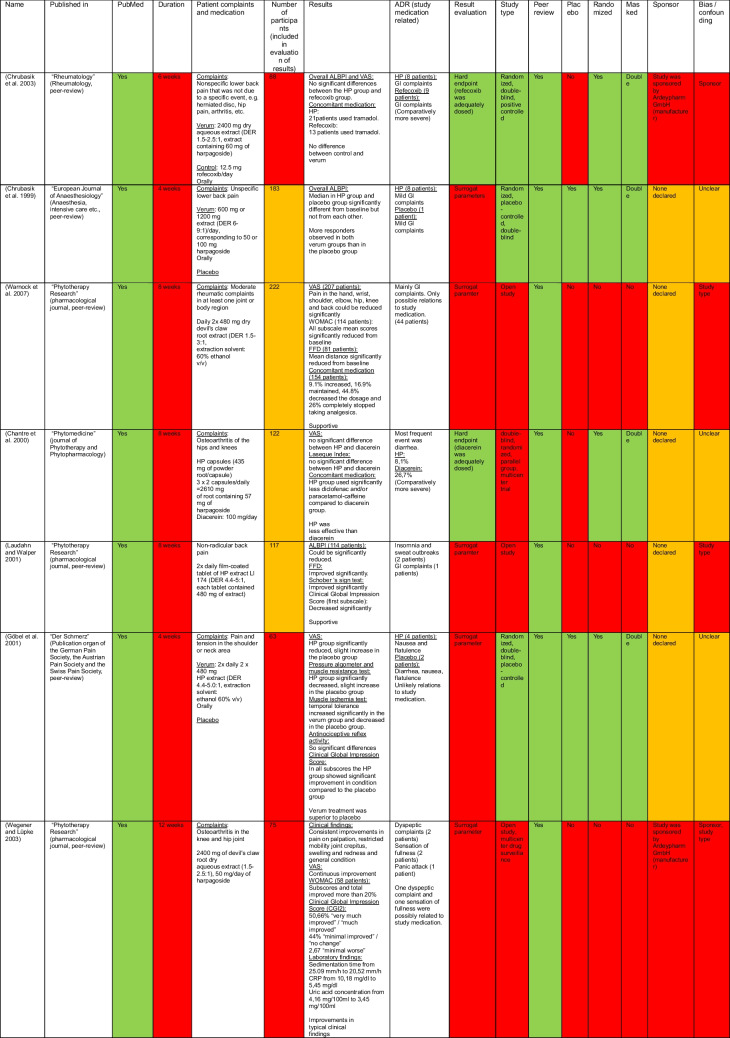
Summary of the analyzed clinical studies. Green, increases the scientific quality. Yellow, factors which cannot be clearly assigned to one or another. Red, decreases the scientific quality

#### Data analysis

The selected studies were analyzed using the following parameters: published in, PubMed, duration, patient complaints and medication, the number of participants included in the evaluation of the results, results, ADR related to the medication used in the study, result evaluation, study type, peer review, placebo, randomization, masking, sponsor, and bias and confounding.

The parameter “published in” was used to check where exactly the study was published, while “PubMed” was used to check whether the study could be found in the database. The “duration,” “patient complaints and medication,” “number or participants included in the evaluation of the results,” “results,” and “ADR related to study medication” were included as stated in the studies.

In “result evaluation,” we determined whether there was a clinically relevant (hard) endpoint and, if so, identified which one, or whether a surrogate parameter, was used. When analyzing the “study type,” we checked which study model was used. Under the analysis items “peer review,” we checked if the study was peer reviewed before publication. We checked whether there was a placebo control group in the studies under the analysis parameter “placebo,” while “randomized” was used to check whether the participants were randomly assigned to treatment or control groups. “Masked” was used to check if the studies were conducted as single blind, double blind, triple blind, or not blinded at all. Under “sponsor,” it was analyzed by whom the study was sponsored or if information on this was provided in the study. Finally, “bias/confounding” was used to analyze which factors may have contributed to bias and or confounding in the studies.

A color system was used in the table to make the analysis clearer. Characteristics of a study that decreases its scientific quality were marked in red. Yellow was used if this characteristic neither clearly contributes to the quality of the study nor decreases it. Green was selected if the scientific quality was increased by the corresponding property.

## Results

### Age limit

**Fig. 4:**
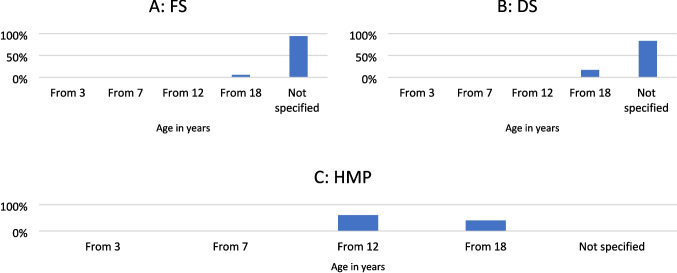
Analysis of the age limit

There was no age limit for 94% of the FS; however, 6% recommended use from the age of 18 (Fig. 4A). Regarding DS, 83% did not specify an age limit, whereas 17% recommended use from the age of 18 (Fig. 4B). As for HMP, 60% indicated use from the age of 12, with 40% recommending use from the age of 18 (Fig. 4C).

### Dosage form

**Fig. 5:**
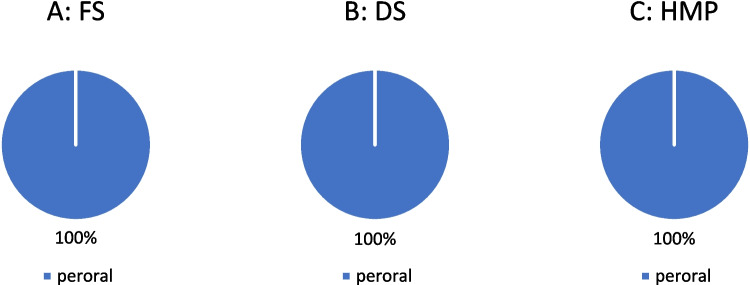
Analysis of dosage form

Figure 5 shows the different dosage forms of the products analyzed. A total of 100% of the FS (Fig. 5A), DS (Fig. 5B), and HMP (Fig. 5C) indicated peroral use.

### Drug-drug interactions

**Fig. 6:**
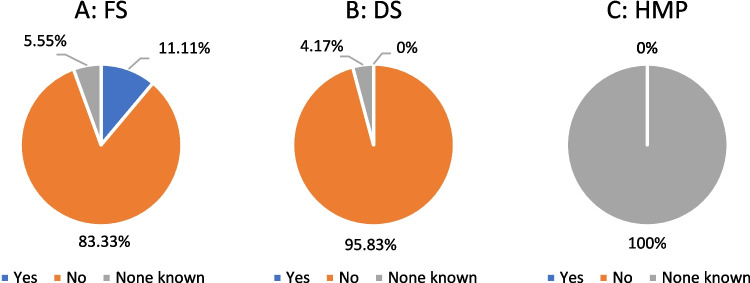
Analysis of indication of drug-drug interactions

A total of 11% of the FS indicated possible drug-drug interactions, while 6% stated that none was known. A total of 83% did not provide any information (Fig. 6A). None of the DS analyzed reported drug-drug interactions, 4% stated that none was known, and 96% gave no information at all (Fig. 6B). A total of 100% of the HMP stated that no drug-drug interactions were known (Fig. 6C).

### Contraindications

**Fig. 7:**
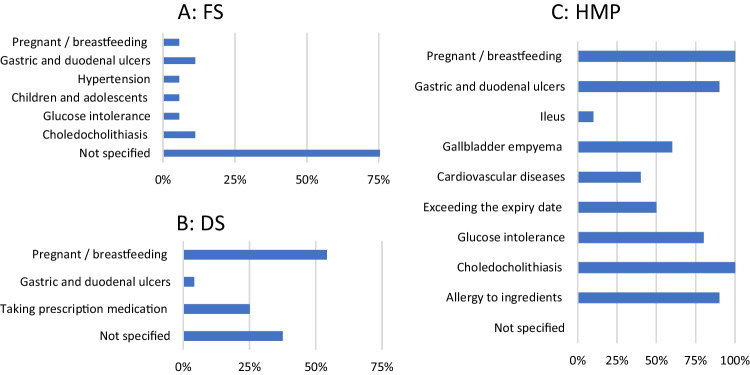
Analysis of contraindications

Regarding contraindications, 78% of FS indicated no contraindications. However, 11% each stated choledocholithiasis and gastric and duodenal ulcers. At 6% each, glucose intolerance, children and adolescents, hypertension, and pregnant or breastfeeding women were the least frequently cited contraindications in this category (Fig. 7A). For DS, 38% did not state any contraindications, while 54% stated pregnant or breastfeeding women and 25% taking prescribed medication. A total of 4% stated gastric and duodenal ulcers as contraindications (Fig. 7B).

In each case, 100% of HMP stated pregnant or breastfeeding women and choledocholithiasis as contraindications. Additionally, 90% stated each gastric and duodenal ulcers and allergy to ingredients. Glucose intolerance was stated by 80%, gallbladder empyema by 60%, exceeding the expiry date by 50%. 40% statet cardiovascular disease and 10% ileus. None of the HMP did not indicate any contraindications (Fig. 7C).

### ADR

**Fig. 8:**
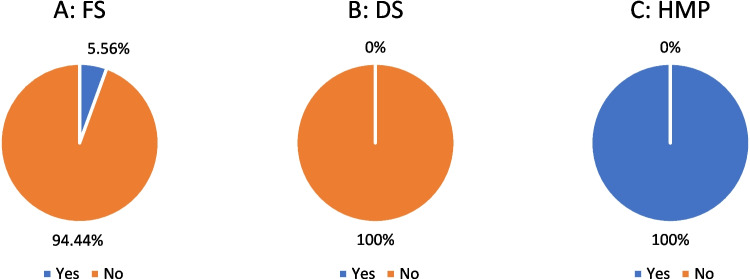
Analysis of ADR

A total of 6% of FS reported, and 94% did not report ADR (Fig. 8A). On the one hand, 100% of DS did not report ADR (Fig. 8B), whereas 100% of HMP did (Fig. 8C).

### RDD

With regard to the RDD, 3 of the 18 FS analyzed, consisting of comminuted or powdered herbal substance for infusion, did not provide any information on the RDD of devil’s claw extract.

**Fig. 9:**
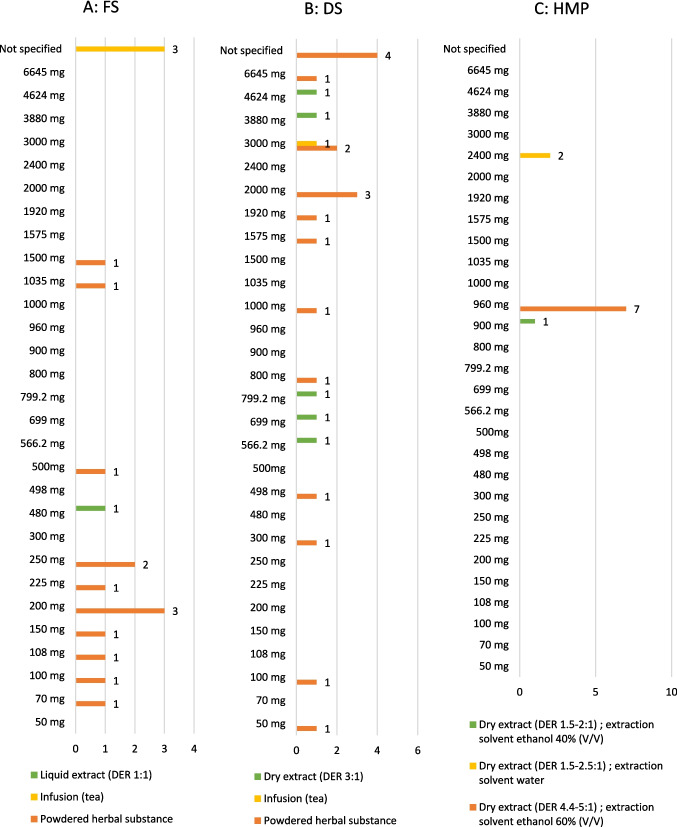
Analysis of the RDD in milligrams of devil’s claw extract

One FS, which was marketed as liquid extract with a DER of 1:1, stated a RDD of 480 mg. A further 2 FS, marketed as a powdered herbal substance, stated 250 mg as the RDD. The others showed a wide variation in dosage (Fig. 9A).

The analysis of a total of 24 DS showed that 4, marketed as powdered herbal substance, gave no information regarding RDD of devil’s claw extract. One DS of this type gave a RDD of 6645 mg, and 3 DS recommended 3000 mg in this regard. One DS, consisting of comminuted or powdered herbal substance for infusion, recommended a daily dose of 4624 mg. Labeled as a dry extract with a DER 1:3, one DS gave a RDD of 3880 mg. In this category, too, the others showed a wide variation in dosage (Fig. 9B). Seven of the 10 HMP analyzed stated an RDD of 960 mg. Two further HMP stated an RDD of 2400 mg and 1 HMP 900 mg.

### Classification

**Fig. 10:**
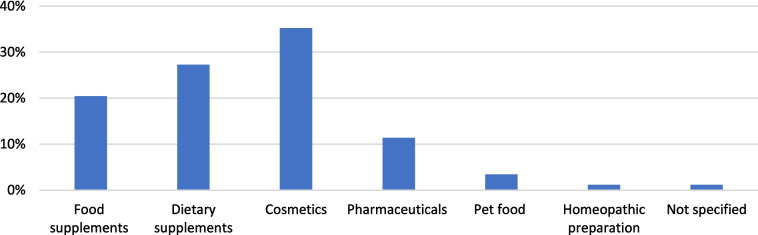
Analysis of the classification

Figure 10 shows that 11% were classified as HMP, 27% as DS, and 20% as FS. A further 35% were cosmetics, 3.41% were marketed as pet food, 1.14% as homeopathic substances, and 1.14% did not provide any information allowing classification.

### Recommended maximum duration of use

**Fig. 11:**
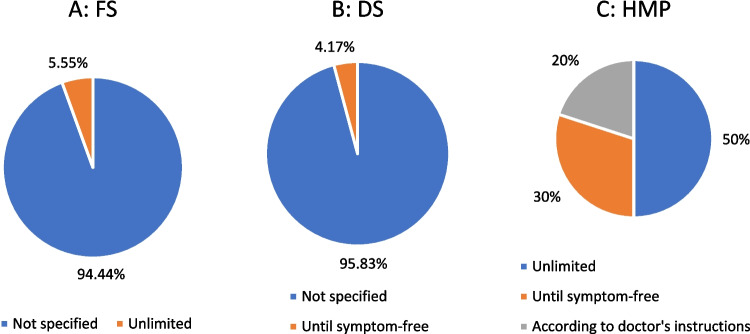
Analysis of recommended maximum duration of use

A total 94% of FS did not specify a maximum duration of use. A total of 6% indicated an unlimited duration of use (Fig. 11A). For DS, 96% did not indicate a maximum duration of use, while 4% recommended use until symptom-free (Fig. 11B). For HMP, 50% gave an unlimited duration of use, 30% recommended use until symptom-free, and 20% recommended use according to medical advice (Fig. 11C).

### Daily prices

**Fig. 12:**
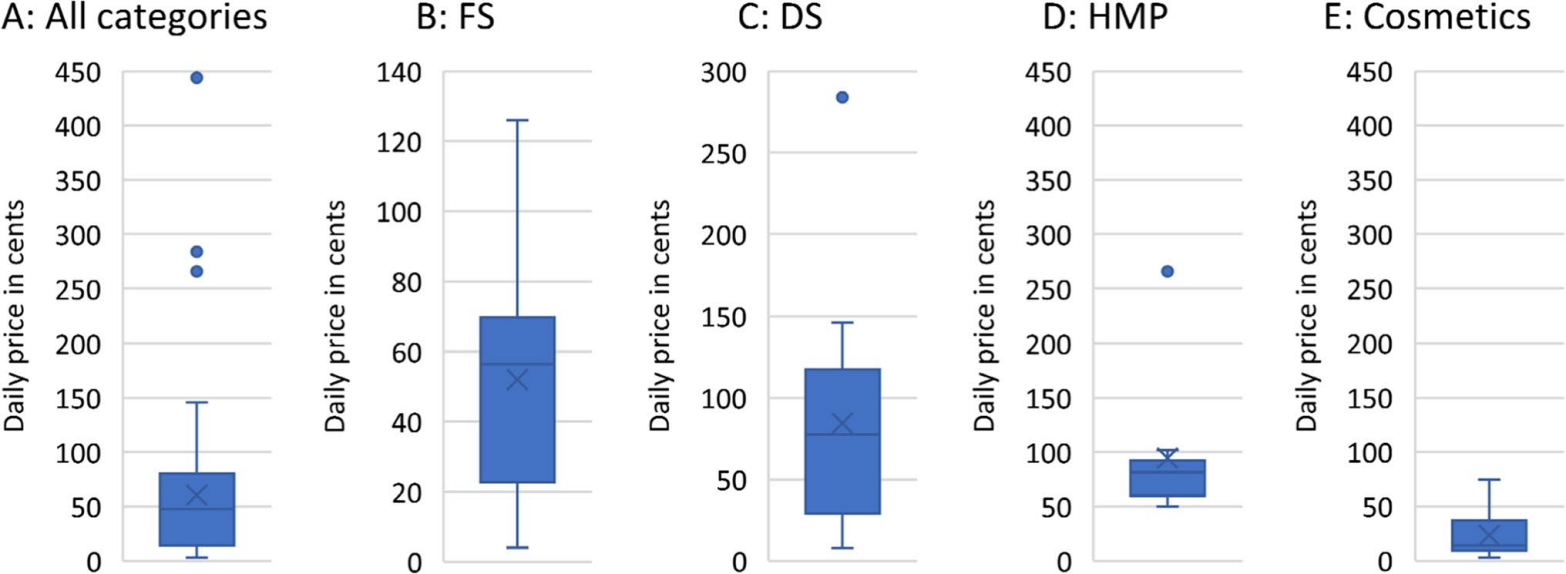
Analysis of the daily prices

Figure 12 shows the broken-down analysis of the daily prices of the three largest classifications of all products analyzed, based on the absolute number. The box corresponds to the area containing the middle 50% of the data. It is delimited by the upper and lower quartiles. The median values are represented by the lines centered in the blue boxes. The crosses represent the mean values. Maximum and minimum values are marked by the upper and lower whiskers respectively. The dots outside the boxes mark the outliers. The mean value for all products was 60.64 cents (Fig. 12A), for FS 52 cents (Fig. 12B), for DS 84.38 cents (Fig. 12C), for HMP 94.5 cents (Fig. 12D), and for cosmetics 23.6 cents (Fig. 12E).

## Discussion

### Legal framework for FS, DS, and THMP

#### Food supplements

FS are regulated by the European Food Safety Authority (EFSA) (https://www.efsa.europa.eu/de/topics/topic/food-supplements, accessed 08.08.2024). Although these products are classified as foodstuffs, they are intended to supplement the normal diet and not to replace it. Unlike medicines, they are not allowed to advertise that they treat or cure certain diseases. Responsibility for quality and safety lies with the manufacturer.

In Germany, only notification of the placing on the market of a new product can be required, for example, by the Federal Office of Consumer Protection and Food Safety (BVL) (https://www.bvl.bund.de/DE/Home/home_node.html;jsessionid=F052971743487B8DDC42CF4E20FD09E2.internet962, accessed December 14, 2024). However, the authority does not examine, evaluate, or issue any authorizations. After notification by the manufacturer, this is forwarded to the Federal Ministry of Food and Agriculture (BMEL) for monitoring. Currently, only the addition of minerals and vitamins and the forms in which they may be used are explicitly regulated. The same applies to substances that may not be contained in FS or are subject to certain regulations. There are no legally defined maximum quantities for ingredients. Furthermore, FS do not have to be able to demonstrate efficacy, and the quantities stated can deviate by up to 50% from the actual quantities contained in the product (https://www.bvl.bund.de/DE/Arbeitsbereiche/01_Lebensmittel/03_Verbraucher/04_NEM/01_NEM_Arzneimittel/NEM_Arzneimittel_node.html#:~:text=Grundsätzlich%20gilt%3A%20Arzneimittel%20sollen%20Krankheiten,verkauft%20werden%20oder%20aufgemacht%20sein, accessed October 18, 2023).

The labeling obligation is limited to the indication of a recommended daily dose in portions as well as information that it should not be exceeded, that the products must be kept out of the reach of children, and that FS should not be used as a substitute for a varied diet. Companies wishing to place a nutrient source on the market that is not included in the list of permitted substances must apply to the European Commission. EFSA then prepares a scientific opinion to evaluate the application to support the European Commission’s decision. Based on EFSA’s work, the Commission regularly reviews and updates the list of vitamins and minerals that may be used in food supplement (https://www.efsa.europa.eu/de/topics/topic/food-supplements, accessed 08.08.2024).

#### Herbal medicinal products

The regulation of herbal medicinal products in the European Union (EU) is the responsibility of the European Medicines Agency (EMA) and is carried out in accordance with the Directive on Traditional Herbal Medicinal Products (2004/24/EC) (https://eur-lex.europa.eu/legal-content/en/ALL/?uri=CELEX%3A32004L0024, accessed December 14, 2024). These medicinal products are authorized as traditional herbal medicinal products (THMP) if there is insufficient evidence of their clinical efficacy.

In Germany, they are subject to the German Medicines Act and must be tested by the Federal Institute for Drugs and Medical Devices, whereby efficacy must be proven and safety standards must be met. The quantities stated may not deviate by more than 5% from the actual quantities, and all ingredient dosages are precisely defined. This allows manufacturers of devil’s claw products who market their products as FS to avoid the stricter and more cost-intensive controls and requirements of HMP (https://www.bvl.bund.de/DE/Arbeitsbereiche/01_Lebensmittel/03_Verbraucher/04_NEM/01_NEM_Arzneimittel/NEM_Arzneimittel_node.html#:~:text=Grundsätzlich%20gilt%3A%20Arzneimittel%20sollen%20Krankheiten,verkauft%20werden%20oder%20aufgemacht%20sein., accessed October 18, 2023).

An important prerequisite for authorization is that the herbal active substance has been used for at least 30 years, including at least 15 years in the EU, and that it is intended for oral use without medical supervision. The long-standing medical use can demonstrate plausibility of efficacy and safety, which means that no clinical tests and trials on safety and efficacy are required in this case as long as sufficient data on these points can be demonstrated.

As a rule, bibliographic data are used to assess safety and efficacy (https://www.ema.europa.eu/en/human-regulatory-overview/herbal-medicinal-products, accessed May 18, 2024). The directive also specifies quality requirements, such as compliance with good manufacturing practice (GMP). In order to obtain marketing authorization, the manufacturer must submit an application to the competent national authority, which examines the application in accordance with the guidelines and standards of the EMA (https://www.ema.europa.eu/en/human-regulatory-overview/herbal-medicinal-products, accessed May 18, 2024).

#### Dietary supplements

In the USA, DS are regulated under the Dietary Supplement Health and Education Act (DSHEA) of 1994 and are subject to oversight by the Food and Drug Administration (FDA). Under DSHEA, DS are considered a subgroup of food. The FDA monitors manufacturers’ compliance with good manufacturing practices (GMP) and can take action to enforce them. Furthermore, manufacturers of DS are required to report serious ADR. Manufacturers of DS do not have to prove the safety of their products or have them tested by the FDA before they market them. Consequently, the FDA can only exercise its control function retrospectively. Furthermore, it can only ban DS if it can prove that they are dangerous. Thus, unsafe or ineffective dietary supplements can be sold freely, while the FDA has limited ability to monitor them. Dietary ingredients include vitamins, minerals, herbs or other botanicals, amino acids, and concentrates, metabolites, or extracts thereof. An ingredient that deviates from this definition is considered a “new dietary ingredient.” The manufacturer of a DS containing a “new dietary ingredient” must submit documentation to the FDA at least 75 days prior to market introduction demonstrating that the product is safe. Manufacturers making structural or functional claims on the label must demonstrate the truthfulness of these claims, as they will not be evaluated by the FDA. The label must also include a disclaimer indicating that the FDA has not evaluated the claims, and that the product is not intended to diagnose, treat, cure, or prevent disease. All food supplements must be labeled as such and list all ingredients. They must be in swallowable form, and other forms are not permitted. Manufacturers must substantiate their claims and must not make claims about the treatment of disease, only claims about nutritional support (Nutrition 2024).

### Comparative analysis of FS, DS, and HMP and the influence of law

#### Classification

Figure 10 shows that only 11% of the analyzed products are marketed as HMP. As the remaining 89% are not classified as medicinal products, they are not subject to pharmaceutical law. From this, it can be concluded that most manufacturers escape the more extensive controls and authorization procedures of the medicinal products, which is particularly evident in the fact that the manufacturers have not provided any information on many analysis parameters.

#### Drug-drug interactions

Figure 6A shows that 83% of the analyzed FS did not provide any information on possible interactions. A similar picture emerged among the DS analyzed, 96% of whom did not provide any information on this subject (Fig. 6B). The fact that the manufacturers did not provide any information on possible interactions in these categories means that this represents a significant health risk for consumers. At the same time, this circumstance can be a potential economic advantage for the manufacturer, as more consumers or customers can be won over to the product, a larger sales market can be created, and more turnover can be generated. In contrast, all HMP stated that there were no known drug-drug interactions (Fig. 6C).

Only limited evidence regarding drug-drug interaction can be found in the literature. In vitro study suggests that devil’s claw inhibits the cytochrome P450 isoenzyme CYP2C9. However, there is only one documented clinical case to date in which an increased bleeding tendency in the form of purpura occurred when devil’s claw and warfarin were taken simultaneously (Williamson et al. 2009). This described interaction between devil’s claw and warfarin was classified as “possible” on Naranjo’s scale, which could indicate a potential inhibition of platelet aggregation by devil’s claw and thus an enhancing effect on warfarin (Patel and Gohil 2008). Nevertheless, the findings are not sufficient to make clear clinical recommendations but should be considered as a possible drug interaction if otherwise unexplained bruising occurs in a patient taking coumarin (Williamson et al. 2009).

#### Recommended maximum duration of use

In contrast to the HMP (Fig. 11C), of which 100% stated a recommended maximum duration of use, it was found that the majority of FS (94%; Fig. 11A) and DS (96%; Fig. 11B) did not indicate such. Something comparable can be seen in Fig. 7 (contraindications), Fig. 8 (ADR), and Fig. 4 (age limit).

Since the consumer has no guideline to orientate himself by, there are two problems. Excluding the question of the effectiveness, the consumer could take it for too short a period, and there would be no effect. The more likely aspect, however, is that this could again result in a potential financial advantage for the manufacturer, as the consumer could take the products over a very long period of time, and this would guarantee the manufacturer a turnover.

Thus, in most cases, the consumers must decide for themselves how long they want to take the product.

#### Dosage form

All FS (Fig. 5A), DS (Fig. 5B), and HMP (Fig. 5C) indicated a peroral administration. Thus, all products comply with the respective legal regulations that they are to be taken orally.

#### Active and additional ingredients

Both HMP (Fig. S5C) and DS (Fig. S5B) listed HP as the only active ingredient, while this was the case for 83% of FS (Fig. S5A). In terms of additional ingredients, FS (Fig. S6A) and DS (Fig. S6B) showed a much more heterogeneous picture. A total of 90% of HMP (Fig. S6C) contained between 9 and 10 additional ingredients. This also reflects the more uniform regulation of HMP compared to FS and DS.

#### Contraindications

Due to a lack of data, HMP is not recommended for pregnant and breastfeeding women. There are insufficient studies on genotoxicity, fertility, pregnancy, and breastfeeding (EMA 2021). The bitter substances HP contains, which stimulate the production of stomach acid, can lead to gastroduodenal ulcers, which is why they are considered a contraindication (ESCOP 2009). Patients with gallstones should also consult a doctor (Weltgesundheitsorganisation 2007). Due to a lack of data, use in children and adults under the age of 18 is not recommended. It is striking that 78% of FS (Fig. 7A) and 38% of DS (Fig. 7B) did not provide any information on contraindications. This represents a considerable risk for consumers. Only 11% of FS cited gastroduodenal ulcers as a contraindication, while 11% mentioned choledocholithiasis, with none of the DS doing so. In line with the regulations, all HMP (Fig. 7C) reported contraindications. Of these, 100% mentioned choledocholithiasis and 90% gastroduodenal ulcers as contraindications.

#### Package sizes and prescription type

All the products analyzed are available over the counter according to the law. Consequently, the potential consumer or patient can access the products very easily. It is noticeable here that all HMP (Fig. S3) were only available in tablet form. FS (Fig. S1) and DS (Fig. S2), on the other hand, were also marketed in liquid and solid form (not in tablet form). It was also noticeable that the package size of FS (Fig. S1C) and DS (Fig. S2C) in tablet form was significantly larger compared to that of HMP (Fig. S3). This means that the consumer can easily obtain the products but in the case of FS and DS must buy significantly larger packs of tablets directly compared to HMP. This represents a potential financial advantage for the manufacturer.

#### RDD

The EMA assessment report on devil’s claw provides an overview of the medical use of the various types of application over the past years and discusses the appropriate indications and RDD in each case (EMA 2021). The information available there was used for the following comparisons. It should nevertheless be mentioned in advance that this information was provided for HMP, but that this at least provides an approximate or orientation value for the RDD of the DS and FS analyzed.

#### Food supplements and dietary supplements

##### Infusion

For herbal substance for tea preparation, the EMA states an RDD of 4500 mg for symptomatic relief of osteoarthritis and 1500 mg for loss of appetite. The FS applicable to this category (Fig. 9A) did not indicate any concentration of devil’s claw extract. In these cases, the devil’s claw was part of an herbal mix, and it was not further specified. This should be viewed critically, as both very low and very high concentrations can be included. One DS (4%) reported an RDD of 3000 mg. However, since in this case no disease-specific scope of application was specified, such a dosage is questionable.

##### Powdered herbal substances

A total of 75% of DS and 72% of FS fell into this category. The EMA specifies an RDD of three times 435 mg for the traditional use of powdered herbal substance, i.e., 1305 mg. Looking at the category of FS (Fig. 9A), it is noticeable that 67% of these were below this, in some cases very significantly. The RDD closest to this value were 1035 mg and 1500 mg, respectively.

The DS category (Fig. 9B) is similarly heterogeneous. The highest RDD in this category was 6645 mg and the lowest 50 mg. The RDD closest to the reference value were 1575 mg and 1000 mg. However, it should also be critically noted that 4 DS (17%) did not provide any information regarding the devil’s claw extract contained. On the one hand, this can be viewed critically regarding consumer safety, but, on the other hand, it may also indicate a potential economic interest on the part of the manufacturers. If, for example, very little of the extract is contained, the consumer would have to consume significantly more to achieve the desired effect, which in turn could represent a potential financial advantage for the manufacturer.

The indication of a high concentration of the extract or the RDD could suggest a stronger effect to the consumer. The fact that there are no legally regulated upper limits or maximum values for FS ingredients in the EU by the EFSA or for DS in the USA by the FDA means that manufacturers have absolute freedom to decide how much of the approved substances they want to put in their products. This must be viewed critically, as no dose–response studies are available and such a strong deviation from the dosages of long-standing medicinal use is considered critical. In this context, however, it must also be mentioned that no toxic effects due to overdose have yet been observed (EMA 2021).

##### Liquid extract (DER 1:1)

One FS (6%), which was classified as a liquid extract with a DER 1:1, indicated an RDD of 480 mg. Regarding these liquid extracts, the EMA assessment report describes a dosage of 1030 mg as RDD. Although this is stated for HMP regarding digestive support, as mentioned above, the significantly lower RDD of FS can be viewed critically.

##### Dry extract (DER 1:3)

One of the DS (4%), with a DER 1:3, was the only one to specify an extraction solvent, 71–81% ethanol. Due to the imprecision of this information, no exact comparison can be made with the information from the assessment report.

For example, for a DER 2.5–4.0:1, with an extraction solvent of ethanol 70% (V/V), the EMA states an RDD of 240 mg for digestive support and for a DER 3–6:1 and an extraction solvent of ethanol 80% (V/V) an RDD of three times 96 mg, i.e., 288 mg.

In any case, the abovementioned DS with an RDD of 699 mg exceeds both references. All other DS also have significantly higher RDD, with 4624 mg as the highest value.

The fact that only one of the corresponding DS indicated an extraction solvent or its proportion, only in an approximate concentration range, makes comparison with the EMA assessment report and its evaluation difficult. Nevertheless, some of the DS showed a multiple of the comparable RDD from the EMA assessment report and should therefore be evaluated critically, if not unsafely.

#### Herbal medicinal products

##### Dry extract (DER 1.5–2:1): extraction solvent ethanol 40% (V/V)

For traditional use, the EMA specifies an RDD of 2–3 times 300–900 mg, i.e., from 600 to 2700 mg. The corresponding HMP (10%) (Fig. 9C) indicated an RDD of 900 mg and was therefore within this reference range.

##### Dry extract (DER 1.5–2.5:1): extraction solvent water

For traditional use in this category, the EMA differentiates between two indications and the corresponding RDD. On the one hand, symptomatic relief of digestive disorders such as dyspepsia and flatulence with an RDD of 2–3 times 100 mg, and on the other hand, the adjuvant treatment of degenerative diseases in the locomotor system with an RDD of 3 times 750 mg-800 mg, i.e. from 2250 to 2400 mg. The indications of the analyzed HMP in this category corresponded (20%) to the last indication mentioned. They stated an RDD of 2400 mg and are therefore still within the reference range.

##### Dry extract (DER 4.4–5:1): extraction solvent ethanol 60% (V/V)

The majority of the analyzed HMP (70%) could be assigned to this category. This exact DER could not be found in the corresponding table in the EMA assessment report but for DER 3–5:1 and extraction solvent ethanol 60% V/V. An RDD of two times 480 mg is given for this, i.e., 960 mg. All corresponding HMP indicated an RDD of 960 mg and thus corresponded exactly to the traditional use described by the EMA.

To summarize the results of the HMP, they reported significantly more homogeneous RDD compared to FS and DS, and that these also corresponded to the statements on the traditional use of the individual categories and the corresponding dosages. This can be explained by the influence of the significantly stricter legal regulations for medicinal products compared to FS and DS.

#### Indication/scope of application and alleged effects

All HMP analyzed had the same indication (Fig. S8C) as well as alleged effect (Fig. S9C), which is also due to the pharmaceutical regulations in the EU. A different pattern can be recognized when looking at the scopes of application of the FS (Fig. S8A) and DS (Fig. S8B). Here, the results are significantly more heterogeneous. In contrast to HMP, FS and DS are not allowed to advertise with indications that they can cure, treat, diagnose, or prevent diseases according to the respective legal basis. However, some FS gave scopes of application that are borderline regarding the regulations mentioned, e.g., 17% diseases of the musculoskeletal system and 6% fever. In comparison, the DS analyzed gave scopes of application for use that did not indicate a specific use for a disease. However, the fact that 17% of the FS (Fig. S8A) and 8% of the DS (Fig. S8B) did not provide any instructions for use must also be viewed critically, as the consumer is not given any information at all in this case and the suggestive effect of the packaging for a possible use is therefore particularly important.

FS and DS are not allowed to advertise medicinal effects, as this is reserved for pharmaceuticals in both the USA and the EU. Thus, although the analysis of the alleged effects of FS and DS showed that 33% of FS (Fig. S9A) and 50% of DS (Fig. S9B) claimed to only have a supportive function for normal bone and joint function, some FS also claimed to have anti-inflammatory (22%) or pain-relieving effects (11%). In comparison, the DS did not report as many critical effects as the FS, but 13% still reported analgesic effects.

#### Analysis of pictograms

The two most frequently depicted pictograms on the analyzed products (Fig. S4) were the devil’s claw itself (36%) and variously depicted forms of a joint (28%). The images of plants and trees together with the increasingly used color green could suggest to the potential buyer that the product would work with natural ingredients. This could possibly lead to associations that there are no artificial ingredients and that there is nothing wrong with “green nature.” The names of the respective manufacturers on the packaging, such as “Nature’s way,” “Garden Organics,” “Secrets of the Tribe,” “Nature’s Answer,” “MegaFood,” or “Irwin Naturals,” could also create an association with a “purely natural” product without “artificial additives” in potential customers. Furthermore, the image of joints and people in motion could create the impression that the product helps with joint problems and contributes to an active lifestyle.

### Parallels to other market studies regarding FS and HMP

However, this situation does not appear to be an isolated case. A similar picture of the market situation was already revealed in a study that focused on ginkgo products for the treatment of dementia and the corresponding market situation (Trabert and Seifert 2023). If one compares the abovementioned study with the results of this study regarding the comparison of FS and HMP, parallels can be identified.

#### Classification

On the one hand, the study on ginkgo products shows that the majority of the products analyzed were declared as FS and significantly fewer as HMP. For reasons of application, the study by Trabert and Seifert showed that the products were either labeled as FS or as HMP (2023). Due to the wide range of possible applications of devil’s claw, in addition to the classifications already mentioned, they were also marketed primarily as cosmetics, which at 35% account for a high percentage of the total number (Fig. 10).

#### ADR

There are also similarities in the information on adverse drug reactions provided by the manufacturers. In the ginkgo study, for example, 91% of the FS did not provide any information on possible ADR, while all the HMP did (Trabert and Seifert 2023). Our results showed that 94% of the FS (Fig. 8A) did not provide any information on possible ADR, whereas all HMP did (Fig. 8C). This crystallizes a comparable value.

#### Drug-drug interactions

A similar picture also emerges for the indication of possible drug-drug interactions. Our results show that 83% of the FS did not report any interactions with other medications or substances (Fig. 6A). All HMP stated that no possible interactions were known (Fig. 6C). In the ginkgo study, the majority of the FS (78%) did not provide any information on possible interactions (Trabert and Seifert 2023). However, both the ginkgo study and our results showed that 100% of HMP provided specific information on this topic.

#### Maximum duration of use

A total of 94% of all FS (Fig. 11A) did not provide any information on this, whereas 100% of MHP (Fig. 11C) did. Likewise, all analyzed HMP in the ginkgo study stated a maximum duration of use, whereas 91% of the FS did not (Trabert and Seifert 2023). This comparison also shows a clear trend, as both our results and those of Trabert and Seifert show that the majority of the analyzed FS did not provide any information on maximum duration of use, leaving the consumer to make this decision on their own (2023). Not specifying or stating an unlimited maximum duration of use could be an indication that manufacturers are increasingly acting in a profit-oriented manner. Extended or permanent use by consumers could generate higher sales. This can be a potential financial advantage for the respective manufacturer. If the consumer takes the product for longer than necessary or continues to take it in the hope of alleviating his current complaints, he establishes the product as a lifestyle product in everyday life and takes it permanently as a preventive measure. However, as the potential customer must buy the product before he can take it, the design of the packaging plays a major role. In these cases, the consumer is not given a guideline to which he can adhere or orientate himself.

#### Daily price

The mean daily price of the FS, which was 52 cents (Fig. 12B), is not particularly high, but it must also be considered that in 94% of cases there was no recommended maximum duration of use (Fig. 11A), and the consumer must therefore decide for himself how long he wants to take the product. All products analyzed are available over the counter according to the law, as depicted in Fig. S4. This broad accessibility implies a wider consumer base and, consequently, potential customers. However, for 65% of the analyzed products (Fig. S1), consumers may find themselves obliged to purchase a pack of 51–100 tablets. A similar picture emerges for the DS. The average daily price for these was 84 cents (Fig. 12C), but in 96% of cases, no recommended maximum duration of use was specified (Fig. 11B), which leads to the problem already described. Furthermore, the box plot graphics (Fig. 12C) show that products classified as DS had the widest price range, viewed from the upper to the lower whisker, followed by FS (Fig. 12B) and cosmetics (Fig. 12E). This contrasts with the MP, which had the smallest price range (Fig. 12D).

This circumstance can again be explained by the legal requirements for FS, DS, and HMP. The German Drug Price Ordinance regulates the prices and price ranges of medicinal products on the German market, which is intended to counteract a strong fluctuation in prices. This can also be seen in the corresponding graph (Fig. 12D). However, the outsider shown in the figure is questionable at this point. The fact that there is no such regulation for DS or FS also explains the fluctuation in their prices, which are thus determined by the free market economy. Regarding FS and HMP, the study by Trabert and Seifert came to a similar conclusion, as they were also able to show that the price range of FS was greater than that of HMP and that this can be explained by the influence of pharmaceutical regulation (2023).

In summary, it can be said that the comparison between the legal regulations for HMP and FS reveals clear differences in their effects. Due to the stricter legislation, HMP are generally more uniform as they are subject to strict regulations. Patients receive important information on use, contraindications, and possible consequences such as ADR or drug-drug interactions. However, many manufacturers evade the strict controls and verification requirements by offering their products as FS instead of complying with the stricter requirements for HMP. This creates information gaps that often leave the consumer alone, as no clear guidelines or recommendations are available. This can lead to uncertainties and risks for consumers. In many cases, this leads to a potential financial benefit for supplement manufacturers as they do not have to undergo the expensive and time-consuming processes of HMP approval and regulation.

### Current state of research

There are many in vitro and in vivo studies (Gxaba and Manganyi 2022), but these are not suitable for assessing possible clinical efficacy. There are also no long-term studies, and toxicological studies have so far only been carried out in vitro or on animals and not on patients, which is why little is known in this area. Table 3 shows the analyzed clinical studies. The color distribution clearly shows that most of the studies are of poor scientific quality, which can also be seen in the analysis of the evidence classes of the studies analyzed (Fig. S7). On the one hand, many of the studies do not have an RCT as a study model, and in some cases, the number of test subjects is clearly too low. Furthermore, all the specified time periods in which the studies were conducted were too short, with the longest being 12 weeks. Chronic illnesses or pain is investigated, and in these cases, longer observation periods are required to obtain meaningful results. Some studies were sponsored, which could have led to bias or confounding. Although the remaining studies did not provide any information on possible sponsorship, they did not actively specify non-sponsorship. The scores used by most studies to assess patients and their state of health always contain a subjective component and are therefore not a purely objective means. On the other hand, the results obtained only correlate with a possible effect of the examined product and do not represent causality. This must also be considered against the background of the predominantly positive results of the studies. In contrast to these characteristics of the studies, which must be critically evaluated, the results of the analyzed studies are consistently positive.

There is a positive publication bias that makes the situation appear better than it is in reality. Leemhuis and Seifert came to similar conclusions in their work analyzing the current market situation for homeopathic products (2024). As our results also show, the studies they analyzed also advocated the use of the corresponding products, and there was also a strong positive publication bias in the studies. However, this circumstance appears to be a problem of greater magnitude, as Easterbrook et al. were able to show that a positive publication bias prevails in the medical literature (1991). Another critical aspect is the fact that according to our PubMed search, 86% of the clinical studies we analyzed are older than 20 years, and the most recent study was published in 2007, meaning that there are in fact no current studies on this topic. This and the already small number of studies clearly underline the declining scientific interest in this topic (Fig. 2) and should give pause for thought. The resulting discrepancy between the lack of clinical studies and the high Google presence could indicate a lack of seriousness of the topic.

## Study limitations and future studies

This study does not aim to provide a detailed analysis of the composition of the HP products included. Rather, our primary aim was to examine the circumstances under which HP products were marketed and to assess the type and amount of information provided by manufacturers to consumers and patients. In addition, we investigated the extent to which manufacturers adhered to recommendations, such as dosing guidelines, and whether consumer safety was ensured or potentially compromised. Another important aspect was the assessment of the consensus of scientific studies, especially regarding their accessibility for potential consumers or patients. In addition, we analyzed possible parallels with other dietary supplements and food supplements to determine whether a broader, potentially global problem could be identified.

Only the information available to the public was used to obtain information on devil’s claw products. Data from package inserts and information published by the manufacturers on their respective products were ultimately used to analyze these. Due to the incompleteness or complete lack of information, this may have led to distortions in this study. No information on sales figures for devil’s claw products on the German market could be found. Furthermore, not all products could be included due to the large quantity available, which limits the generalizability and transferability of the results. For future studies, it would be interesting to analyze all preparations offered on the EU and US markets. No results regarding embryotoxic effects could be found on embryoTox (https://www.embryotox.de, accessed 01.06.2025). More clinical long-term studies focusing on potential side effects, toxicity, and teratogenicity would be desirable, as they could potentially contribute to consumer safety.

## Conclusion

Devil’s claw products labeled as FS and DS have large gaps in the data available to the consumer, especially when compared to HMP. This is mainly due to the current legal situation regarding regulation and control. Due to the manufacturers’ great freedom of action, they can in many respects arbitrarily decide how much information they want to disclose about their product. Consumer protection should also be questioned in this context, as the frequent failure to provide information such as age limit, maximum duration of use, drug-drug interactions, contraindications, and ADR can lead to negative consequences for the consumer. The RDD of FS and DS, which were significantly higher or lower than those traditionally used in the EU, should also be viewed critically. In contrast, all HMP corresponded to these values. A similar pattern was observed in the study by Trabert and Seifert regarding ginkgo products (2023). Comparing vitamin A products in Germany and the USA, Rathmann and Seifert (2024) showed that many of the FS and DS examined did not meet the legal requirements for warning labels. Manufacturers often did not adhere to the daily doses recommended by the Bundesinstitut für Risikobewertung and the FDA. In many cases, products were offered with vitamin A concentrations that were significantly higher than the recommended values. In addition, the ingredients of food supplements varied greatly, which can lead to unintentional under- or overdosing and thus compromise consumer safety.

This suggests that the issues we identified regarding consumer information with DS and FS are not isolated incidents but rather indicative of a more widespread problem within this sector. The findings highlight potential systemic challenges in the regulation, quality assurance, or transparency associated with dietary supplements. Such parallels underscore the need for more comprehensive studies and regulatory oversight to address these recurring concerns and ensure consumer safety and product reliability across the industry. The manufacturers of FS and DS appear to be primarily pursuing financial interests, which is also reflected in the wide price ranges of these products. To ensure greater safety for consumers, appropriate legal changes would have to be made that oblige manufacturers to provide more detailed information about their products and require them to carry out appropriate checks. By giving the competent authorities, FDA and EFSA, more possibilities and scope for action, they could exercise their control functions more adequately and thus contribute to consumer protection.

The quality of the clinical studies used is poor. Many have too few participants or methodological weaknesses (Fig. 3). In addition, the well-known large placebo effect in the treatment (Zhang 2019) of this disease influences the results, which means that the evidence of efficacy for a clear indication for use is insufficient. Many differences were found in the products studied, including aqueous or ethanolic extracts, various DER, and powdered roots. Similarly, the dose administered varied, either in milligrams of harpagoside or milligrams of herbal substance. In view of these circumstances, which make a direct comparison of the results difficult, it is not possible to adequately assess the effects of devil’s claw. More standardized study models such as RCT, conducted based on objective parameters, are needed to obtain a more meaningful overall picture. In addition, more clinical studies with larger numbers of participants and long-term studies are needed to be able to investigate possible, yet unknown adverse effects or interactions. In summary, it should be noted that the increasing public interest in this topic does not correlate with the scientific or study situation, and, due to this clear discrepancy, the seriousness of the topic should be questioned.

## Supplementary Information

Below is the link to the electronic supplementary material.

Supplementary file1 (DOCX 1101 KB)

## Data Availability

All source data for this work (or generated in this study) are available upon reasonable request.

## Abbreviations

ALBPI: Arhus low back pain index
ADR: Adverse drug reaction
DER: Drug-extract ratio
DM2: Diabetes mellitus type 2
DS: Dietary supplement
EFSA: European Food Safety Authority
EU: European Union
EMA: European Medicines Agency
FDA: Food and Drug Administration
FFD: Finger floor distance
FS: Food supplements
HMP: Herbal medicinal products
HP: *Harpagophytum procumbens*
RCT: Randomized controlled trial
RDD: Recommended daily dose
USA: United States of America
VAS: Visual analog scale
WOMAC: Western Ontario and McMaster Universities Osteoarthritis Index
