# Supplementary material for: Devil’s claw (Harpagophytum procumbens): is the buzz in Google justified?
Source: Naunyn Schmiedebergs Arch Pharmacol. 2025 Mar 5;398(8):10823–42. doi: 10.1007/s00210-025-03974-7 (PMC12350520; doi:10.1007/s00210-025-03974-7)
Supplement: Supplementary file 1 — Supplementary file1 (DOCX 1101 KB) [file 210_2025_3974_MOESM1_ESM.docx]

**Devil’s claw (*Harpagophytum procumbens*): Is the buzz in google justified?**

**Finn Erik Bargsten, Roland Seifert**

Institute of Pharmacology

Hannover Medical School

Carl-Neuberg-Str. 1

D-30625 Hannover, Germany

Correspondence: [seifert.roland@mh-hannover.de](mailto:seifert.roland@mh-hannover.de)

**Keywords**

*Harpagophytum procumbens*, devil’s claw, product analysis, study analysis

**Supplemental figures**

Figure S 1 shows the package sizes of FS in solid, liquid and tablet form.


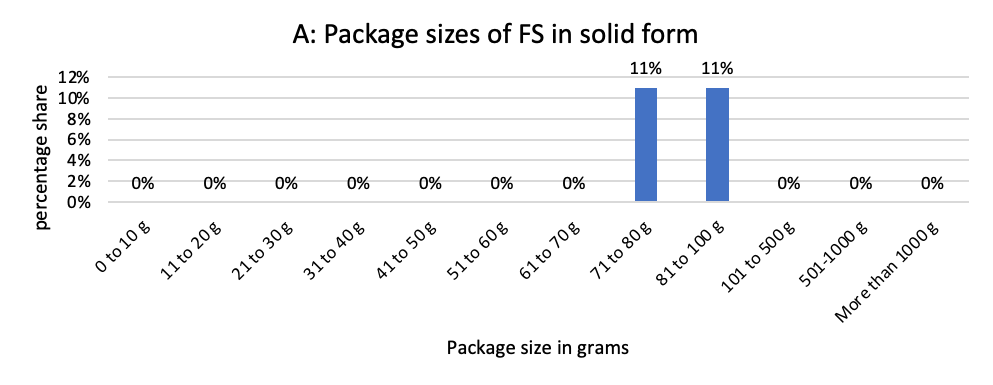

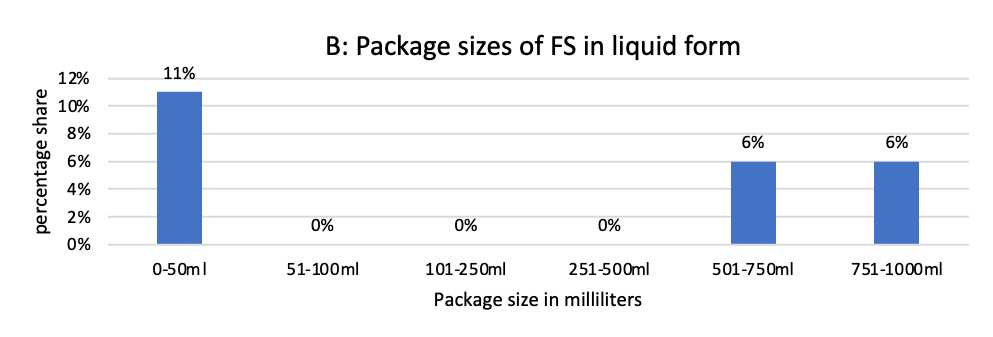

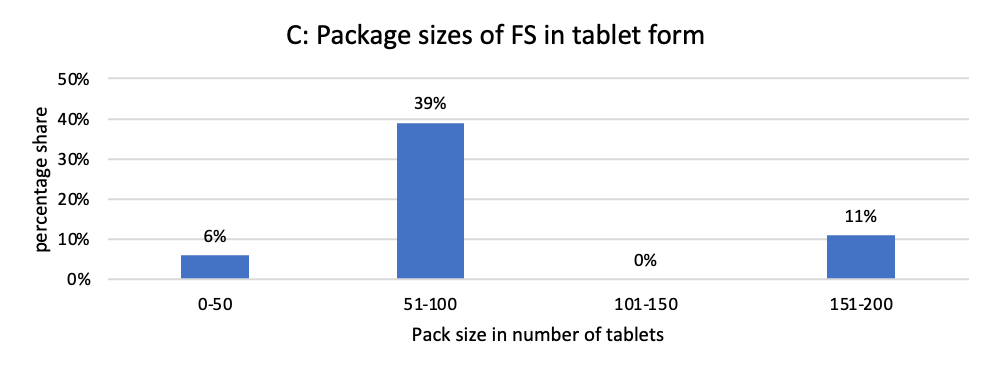


Figure S 2 shows the package sizes of DS in solid, liquid and tablet form.

Figure S 3 shows the package sizes of HMP in tablet form.

Figure S 4 shows that the pictograms on the packaging.

Figure S 5 shows the active ingredients of FS, DS and HMP.


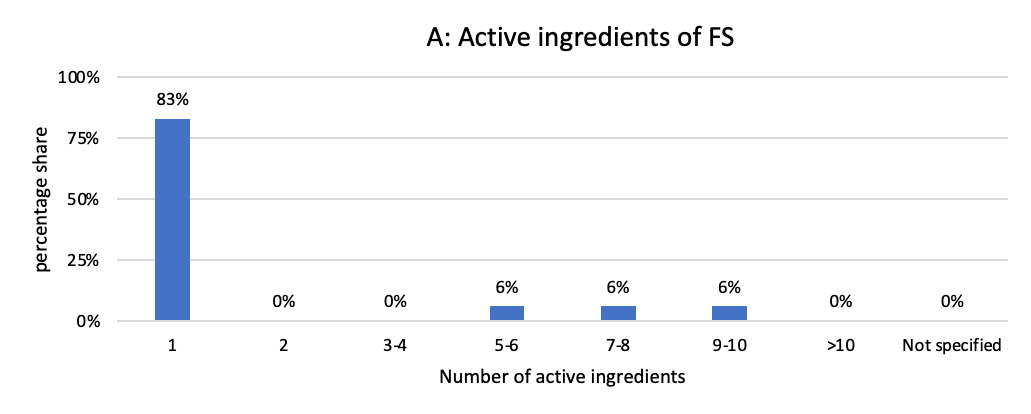

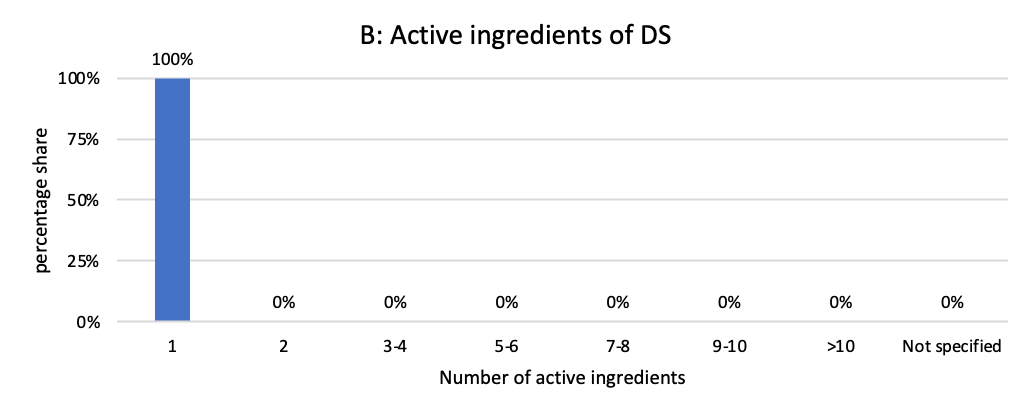

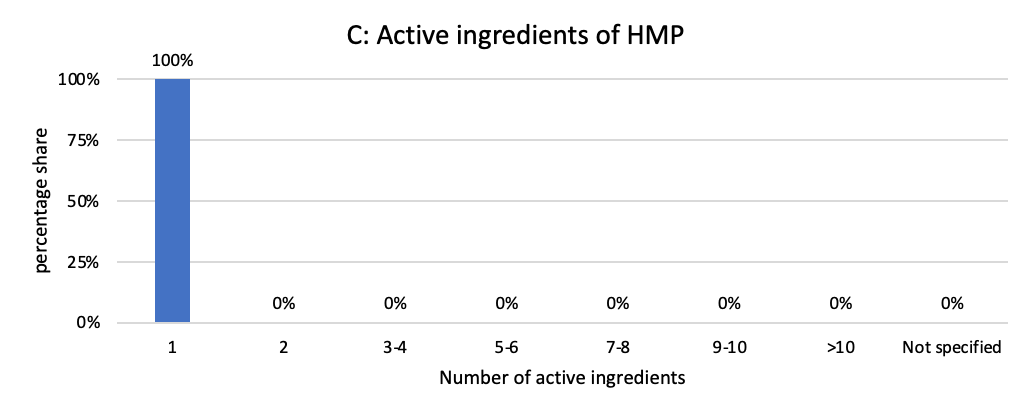


Figure S 6 shows the additional ingredients of FS, DS and HMP.


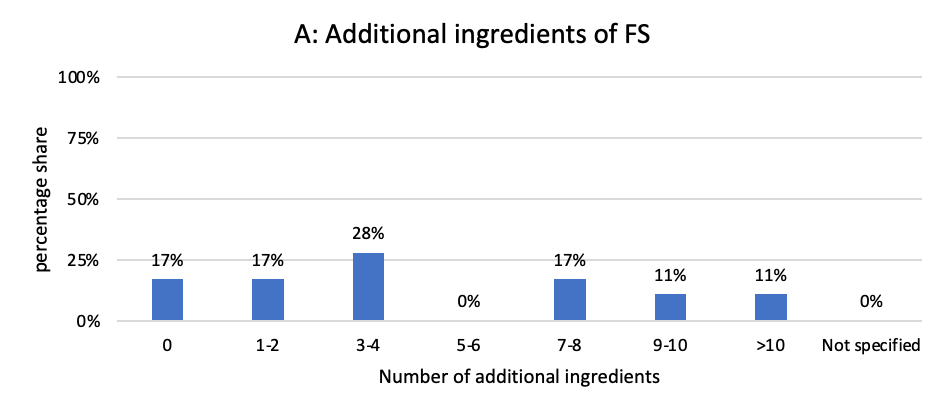

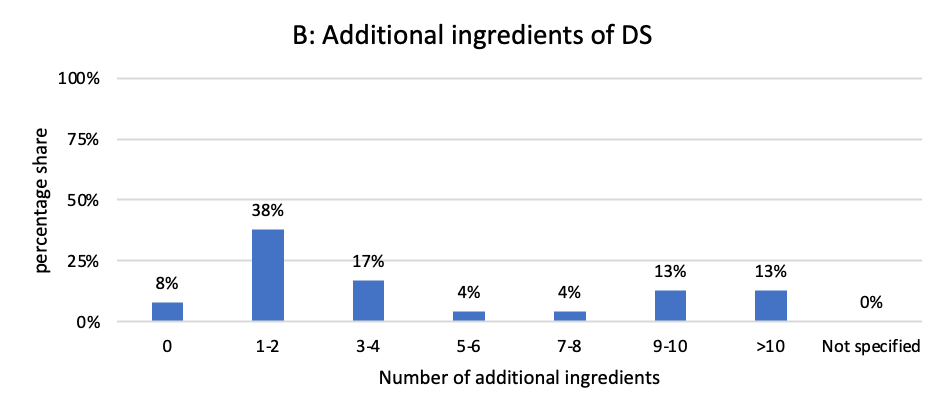

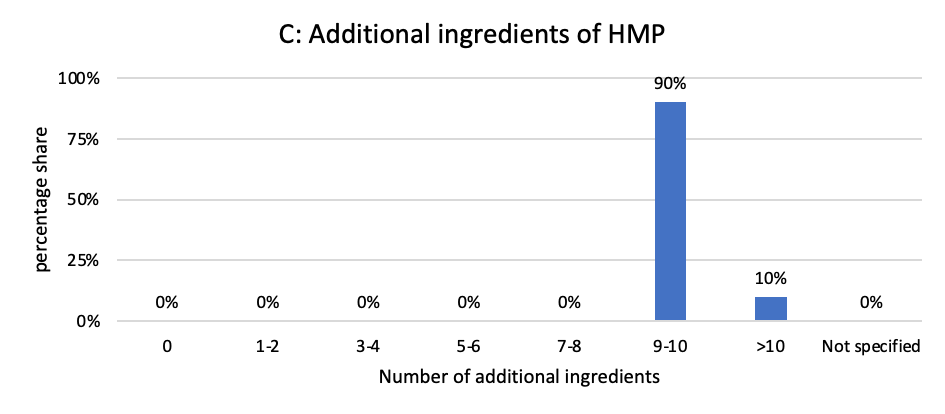


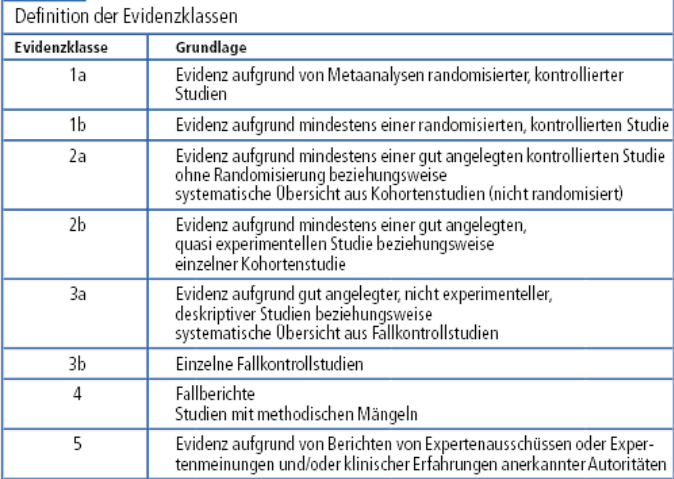


Table S 1 shows the definitions of the different evidence classes.

<https://www.aerzteblatt.de/archiv/48444/Evidenzbasierte-Medizin-am-Beispiel-der-diabetischen-Retinopathie>, accessed 10.18.2023

Figure S 7 shows the studies analyzed in this paper divided into evidence classes.

Figure S 8 shows the scopes of application of FS and DS and the indications of HMP.


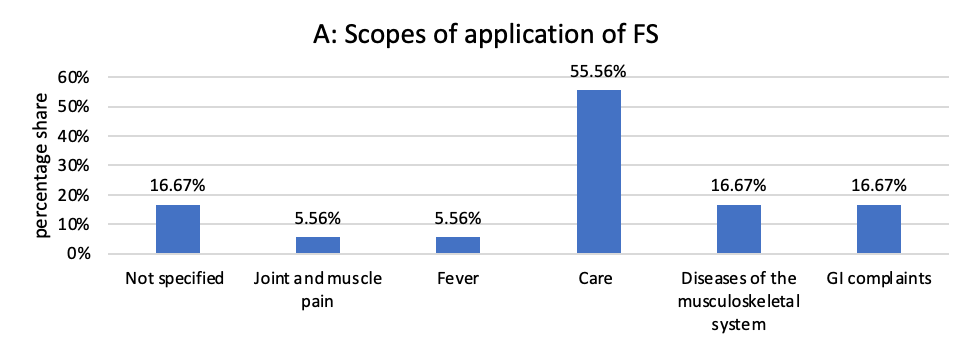

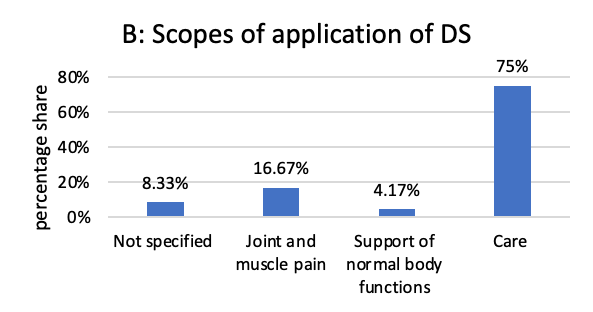

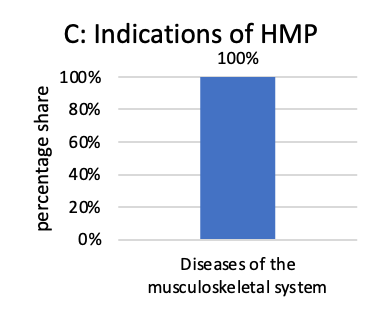


Figure S 9 shows analysis of the alleged effects of FS, DS and HMP.


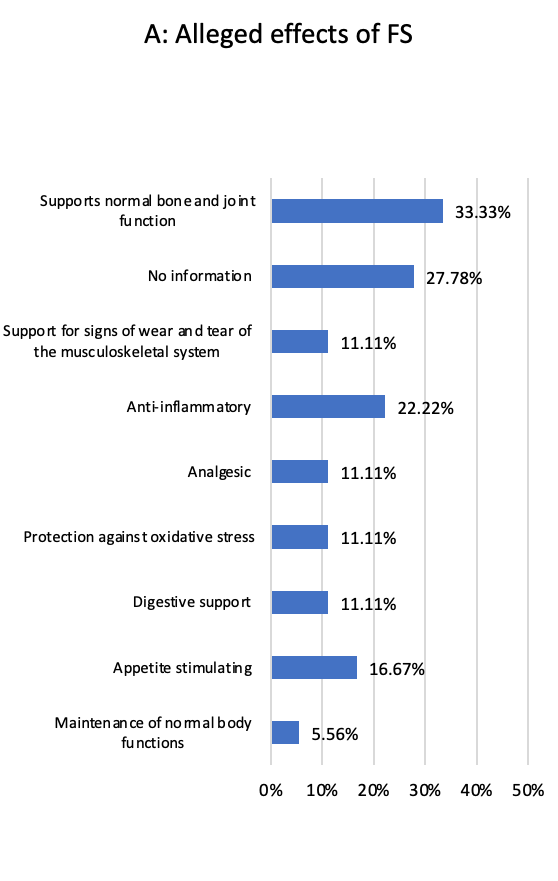

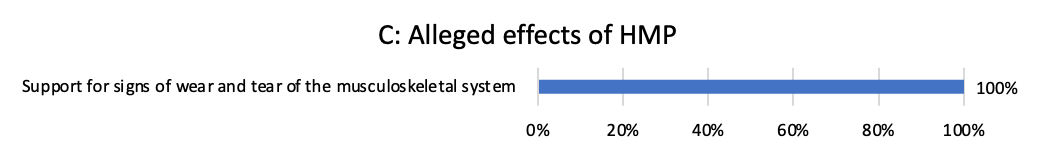

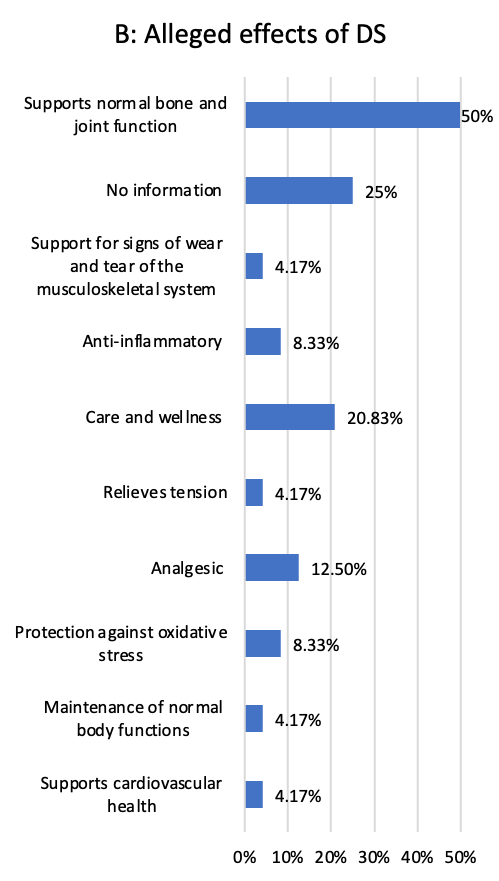


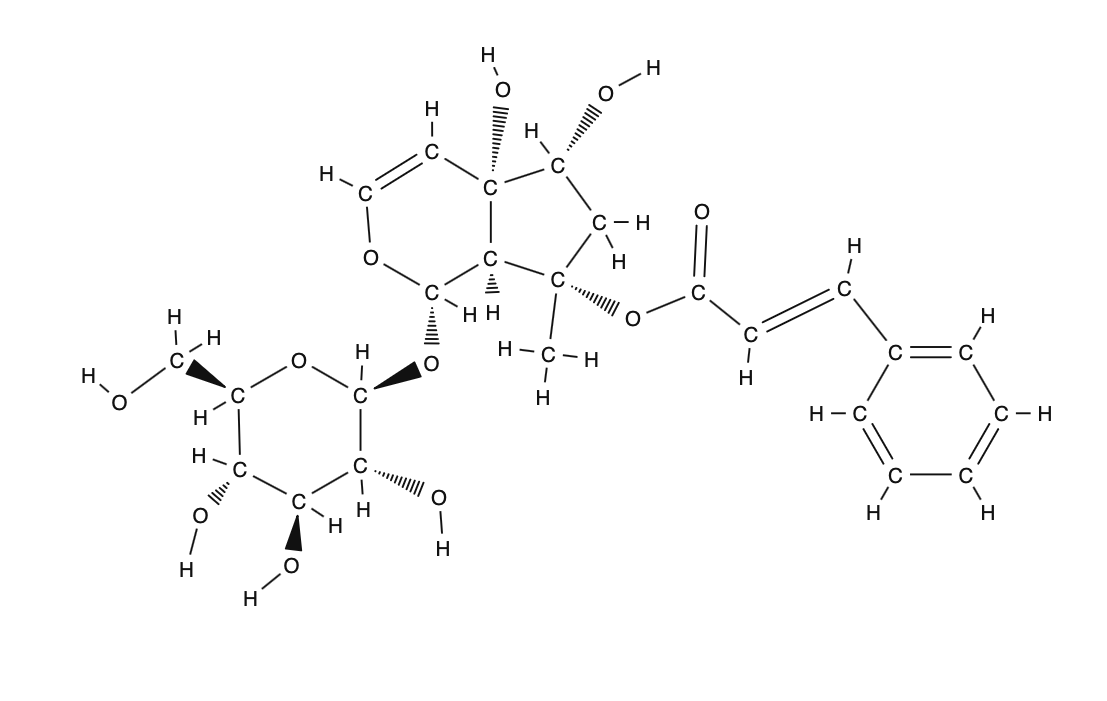


*Figure S 10 shows the chemical structure of harpagoside.*


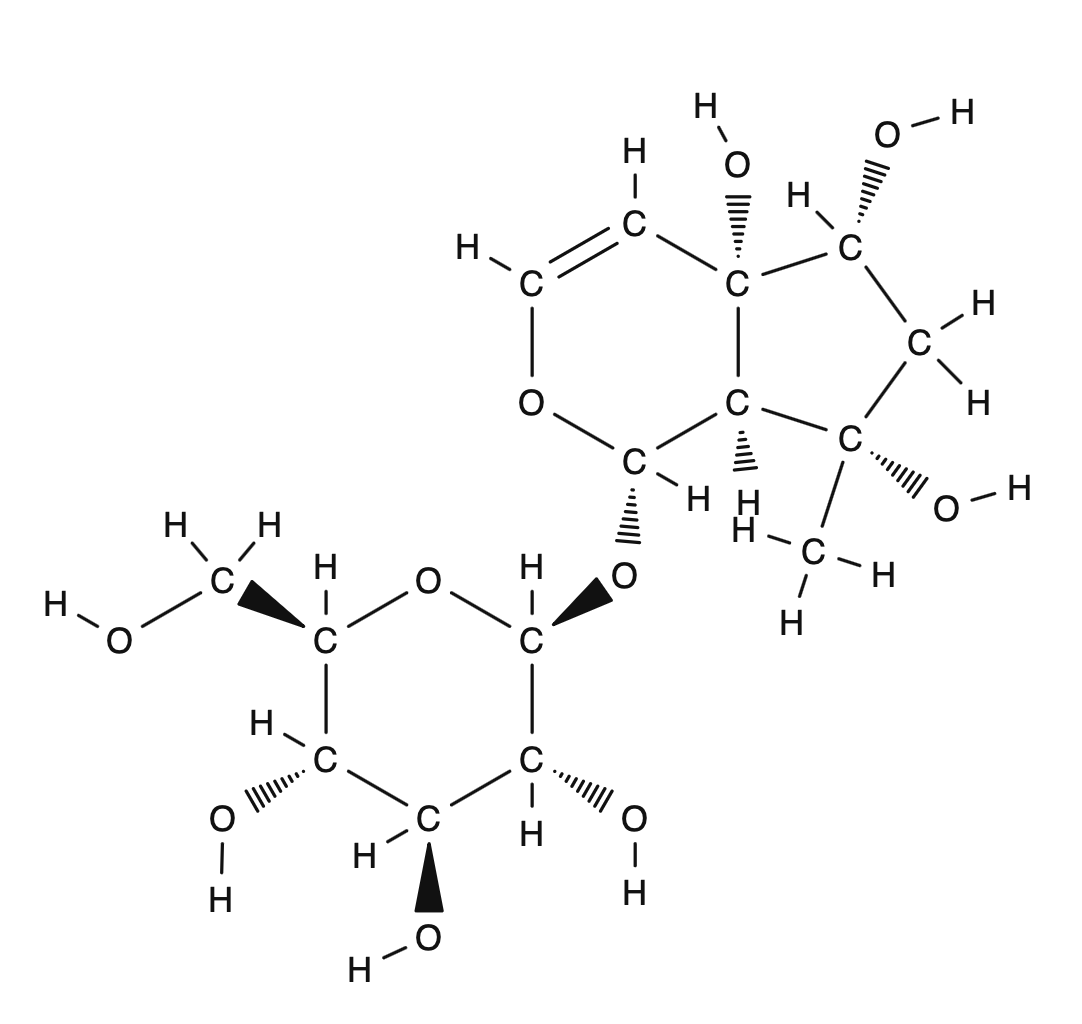


*Figure S 11 shows the chemical structure of harpagide.*
